# *Mycoplasma synoviae* lipid-associated membrane proteins identification and expression changes when exposed to chicken cells

**DOI:** 10.3389/fvets.2023.1249499

**Published:** 2023-11-02

**Authors:** Duoduo Si, Jialin Sun, Lei Guo, Fei Yang, Jidong Li, Shenghu He

**Affiliations:** ^1^College of Animal Science and Technology, Clinical Veterinary Laboratory, Ningxia University, Yinchuan, China; ^2^Ningxia Xiaoming Agriculture and Animal Husbandry Co., Ltd., Yinchuan, China

**Keywords:** *Mycoplasma synoviae*, proteome, bioinformatics analysis, lipid-associated membrane protein, transcript analysis

## Abstract

*Mycoplasma synoviae* is a significant cause of respiratory disease and synovitis among chickens, and has an adverse economic impact on broiler breeding efforts. The present study was designed to develop a systematic understanding of the role that *M. synoviae* lipid-associated membrane proteins (LAMPs) may play in the virulence of this pathogen. Bioinformatics tools were used to identify 146 predicted membrane proteins and lipoproteins in the *M. synoviae* proteome. Then, Triton X-114 was used to extract LAMPs that were subsequently identified via LC–MS/MS. This approach enabled the detection of potential LAMPs, and the top 200 most abundant proteins detected using this strategy were subject to further analysis. *M. synoviae* cells (100 MOI) were exposed to chicken fibroblasts (DF-1) and macrophages (HD-11) in a 1:1 mixed culture. Analysis of LAMP transcripts identified 72 up-regulated LAMP genes which were analyzed in depth by bioinformatics. GO analysis revealed these genes to be enriched in the nucleotide binding, sulfur amino acid transmembrane transporter activity, tRNA binding, rRNA modification, and transition metal ion transport pathways. Moreover, KEGG enrichment analysis suggested that these genes were enriched in the biosynthesis of secondary metabolites, carbon metabolism, glycolysis/gluconeogenesis, and nitrogen metabolism pathways.

## Introduction

*Mycoplasma synoviae* is a bacteria that is related to smaller Gram-positive bacteria and that is a common cause of infectious disease among poultry and other birds, causing characteristic Mycoplasma synovialis disease (1). This disease exhibits a high degree of tissue tropism, with arthritic-type disease impacting the joints, keel bursa, synovial sac, and tendon sheaths, whereas the respiratory type impacts the airways and air sac. Both vertical and horizontal transmission of *M. synoviae* have been reported, contributing to extensive morbidity and mortality that result in major economic losses for the global poultry industry.

Bacterial outer membrane vesicles (OMVs) can activate macrophages and splenic mononuclear cells from chickens, primarily through the effects of membrane proteins and lipopolysaccharides (2). Mycoplasma infection necessitates the initial adhesion and colonization of epithelial sites, and lipid-associated membrane proteins (LAMPs) are required for this adhesion (3), promoting inflammation through the binding of host receptor proteins (4). Members of the *Mycoplasma hyorhinis* variable lipoprotein (Vlp) family can interact with Mhr, plasminogen, and the host extracellular matrix (ECM) in the context of cellular adhesion, with differences in Vlp size likely playing a role in the regulation of these interactions (5). *Mycoplasma bovis* membrane proteins mainly include heat shock proteins, glycoproteins and lipoproteins, which can stimulate cellular immunity and humoral immune responses. And the variable membrane protein improves the ability of *M. bovis* to evade host immune defenses (6). The membrane-associated cytotoxic nuclease MGA_0676 derived from *M. gallisepticum* plays an essential role in the nuclear translocation of this bacteria and the induction of apoptotic death in chicken cells (7).

The *M. synoviae* genome is 0.766–0.848 Mb in size, with a median total length of 0.804397 Mb, a GC content ranging from 27.90–28.40% (median: 28.25%), harboring 635–686 protein-coding genes (median protein count: 655). Studies of *Mycoplasma*-associated LAMPs conducted to date have been relatively limited, although molecular chaperone DnaK, glycerophosphodiester phosphodiesterase (GDPD), phosphoglycerate kinase (PGK), lactate dehydrogenase (LDH), FAD-dependent oxidoreductase (NOX), dihydrolipoamide dehydrogenase (DLD), and methylenetetrahydrofolate dehydrogenase (MTHFD) have been determined to be distributed in the cytosol and membrane fractions of *M. synoviae,* with slightly higher DnaK, GDPD, PGK, and LDH levels in the membrane relative to the cytosol, whereas these other proteins exhibit the opposite trend (5, 8–15). NOX, fructose-bisphosphate aldolase (FBA), and DLD have also been reported to play a role in adhesion in the context of *M. synoviae*-mediated infection of chicken cells. Proteins encoded by variable lipoprotein vlhA genes facilitate attachment to sialic acid receptors on host cells and indirectly contribute to synovial sialidase gene sequence diversity.

LAMPs as major structures of bacteria interaction with host cells, could trigger the immune system, modulate apoptosis and cell adhesion (16–18). Some LAMPs in *mycoplasma* have been characterized, such as P40, P50 have been implicated as important adhesion molecules in *mycoplasma* infection, these proteins also have been studied as antigens for vaccine candidates and as targets for antimicrobial drugs (19, 20). Systemic and detailed studies of *M. synoviae* LAMPs have the potential to better support current understanding of the infectious mechanisms whereby this pathogen functions, offering the opportunity to develop novel inhibitors that can specifically target certain proteins in a manner that may better support the control and treatment of *M. synoviae-*associated disease. Here, analysis of highly abundant LAMPs were conducted, and changes in these LAMP profiles following host cell infection were characterized. Together, the results of this study offer a strong foundation for researchers studying the mechanisms of *M. synoviae* pathogenesis and seeking to treat associated diseases.

## Materials and methods

The *M. synoviae* strain WVU 1853 was obtained from the ATCC (1853™). Mycoplasma were cultured in modified Frey medium (Zhonghai Biotech, Beijing, China) to stationary phase in a 37°C, 5% CO2 incubator, with the color change of the medium being used to detect this shift in metabolic activity prior to LAMP collection.

DF-1 chicken embryo fibroblasts and HD-11 chicken macrophages were obtained from the Shanghai Institute of Biochemistry and Cell Biology (SIBC, Shanghai, China) and cultured in DMEM (Thermo Scientific, United States) containing 10% heat-inactivated fetal bovine serum (FBS; Biological Industries, Israel) and 100 IU/mL penicillin in a humidified 37°C 5% CO2 incubator. The morphological characteristics of these cells were visualized via inverted light microscope (Leica DM IL LED and LAS X software).

### Proteomic analysis

The sequences of *M. synoviae* were downloaded from the NCBI database (www.ncbi.nlm.nih.gov/; ID: NZ_CP011096.1) and 663 proteins were analyzed. Subcellular localization analysis were performed with PSORTb^1^ according to gram-negative and gram-positive strain parameters, respectively. Non-classically and classically secreted proteins were, respectively, identified with the Secretome P 2.0 server^2^ and the SignalP 4.1 server.^3^ Transmembrane helical region predictions were performed with TMHMM.^4^ Predictions of lipoproteins were performed with LipoP – 1.0.^5^ The number of LAMPs based subcellular localization and lipoproteins prediction were compared with flowing LAMPs results.

### LAMP extraction

Triton X-114 was used to treat *M. synoviae* cells in order to isolate LAMPs as detailed previously (21). Briefly, a 0.9 mL cell suspension was combined with 0.1 mL of 20% (v/v) Triton X-114, followed by incubation for 2 h on a rotator at 4°C. Insoluble material was then removed by centrifuging samples (10 min, 10,000 × g, 4°C), with supernatants being transferred into new tubes that were warmed for 5 min at 37°C for phase separation, followed by centrifugation (5 min, 10,000 × g). The upper aqueous phase was then discarded, while the 0.9 mL of TS buffer (50 mM Tris-Cl, 0.15 Nacl, PH 8.0) was added to the Triton X-114 phase, and samples were processed again as above. Then, 9 volumes of methanol were added to precipitate lipoproteins in this Triton X-114 phase, after which the concentration of proteins in these Triton X-114 phase.

### Enzymatic protein hydrolysis

One milliliter of the prepared sample was then subject to further processing. A sample containing 100 μL of SDT lysis buffer [4% SDS, 100 mM DTT, 100 mM Tris HCl] was incubated for 5 min in a boiling water bath, cooled to room temperature, centrifuged, and the supernatant fraction was transferred into a fresh tube. An appropriate volume of iodoacetic acid was then added to a final concentration of 50 mM, and sample was incubated for 20 min at room temperature while protected from light. Then, 400 μL of methanol, 100 μL of chloroform, and 300 μL of ddH2O were added to these tubes, followed by further centrifugation (1 min, 9,000 x g). The upper phase was then removed from these triphasic samples, while the middle and lower phases were retained and mixed with 300 μL of methanol. The sample was then centrifuged (2 min, 9,000 × g), supernatants were removed, and a protein pellet was transferred into a fume hood for air drying. Then protein fraction was resuspended in a solution containing 4 μg trypsin in 400 μL NH4HCO3 buffer (2 mM NH4HCO3) followed by incubation for 16–18 h at 37°C. Following enzymatic hydrolysis, these peptides were desalted with the C18 Stage Tip and vacuum dried. Peptides were subsequently reconstituted using 0.1% formic acid, and peptide concentrations were determined for LC–MS/MS analysis based on OD280 values.

### LC–MS/MS analysis

Appropriate peptide samples were selected for chromatographic separation with an EasynLC 1,200 chromatographic system (Thermo Scientific) using buffers A (0.1% formic acid) and B (0.1% formic acid, 80% acetonitrile). The chromatographic column was equilibrated with 100% buffer A, after which the sample was added to the trap column for linear gradient separation at a 300 nL/min flow rate with the following settings: 0–3 min, 2–8% B; 3–98 min, 8–28% B; 98–108 min, 28–40% B; 108–110 min, 40–100% B; 110–120 min, 100% B. A Q-Exactive HF-X mass spectrometer (Thermo Scientific) was then employed for data-dependent acquisition (DDA) mass spectrometry for 120 min. The mass spectrometry database was searched using the MaxQuant 1.6.1.0 program with reference to the *M. synoviae* proteome in the Uniprot Protein Database.

### Bioinformatics analysis of LAMPs

Potential virulence-associated LAMPs were identified by searching for potential orthologous proteins in the Virulence Factor Database,^6^ while the cytotoxic potential of these proteins was evaluated with BTXPred tools. The PHI-base database was used for pathogenic analysis.

### Infection experiments

For *in vitro M. synoviae* infection experiments, DF-1 and HD-11 cells were cultured at a 1:1 (10^5^, 10^5^) ratio in 6-well plates, and monolayers were infected with *M. synoviae* in the mid-exponential phase of growth following resuspension in DMEM at a multiplicity of infection (MOI) of 100. Following incubation for 8 h at 37°C in a 5% CO2 incubator, supernatants were harvested, cells were rinsed three times with sterile PBS, and these fractions were combined with the supernatants. This mixture was then centrifuged (20 min, 5,000 × g, 4°C), and the pellet was collected for RNA extraction using TRIzol based on standard procedures. A parallel experiment was conducted in triplicate.

### RNA-Seq analysis of LAMPs

An RNA-Seq approach was used to explore changes in *M. synoviae* LAMP expression following exposure to DF-1 and HD-11 cells. Eleven candidate genes were used for the verification of gene expression by qRT-PCR. A parallel experiment was conducted in triplicate. The primer information is listed in Supplementary Table 1. Differentially expressed genes (DEGs) were analyzed with Omicshare.^7^ Gene Ontology (GO) enrichment analysis were additionally conducted with Omicshare,^8^ with a small *p*-value corresponding to more significant GO term enrichment. The KEGG database^9^ was further used to analyze the pathways in which differentially expressed LAMPs were enriched, which a protein–protein interaction (PPI) network for these differentially expressed LAMP genes was constructed with STRING^10^ and visualized using the Cytoscape software.

## Results

### Predictive analysis of the localization and secretion of lipoproteins based on the *Mycoplasma synoviae* genome

**Figure 1:**
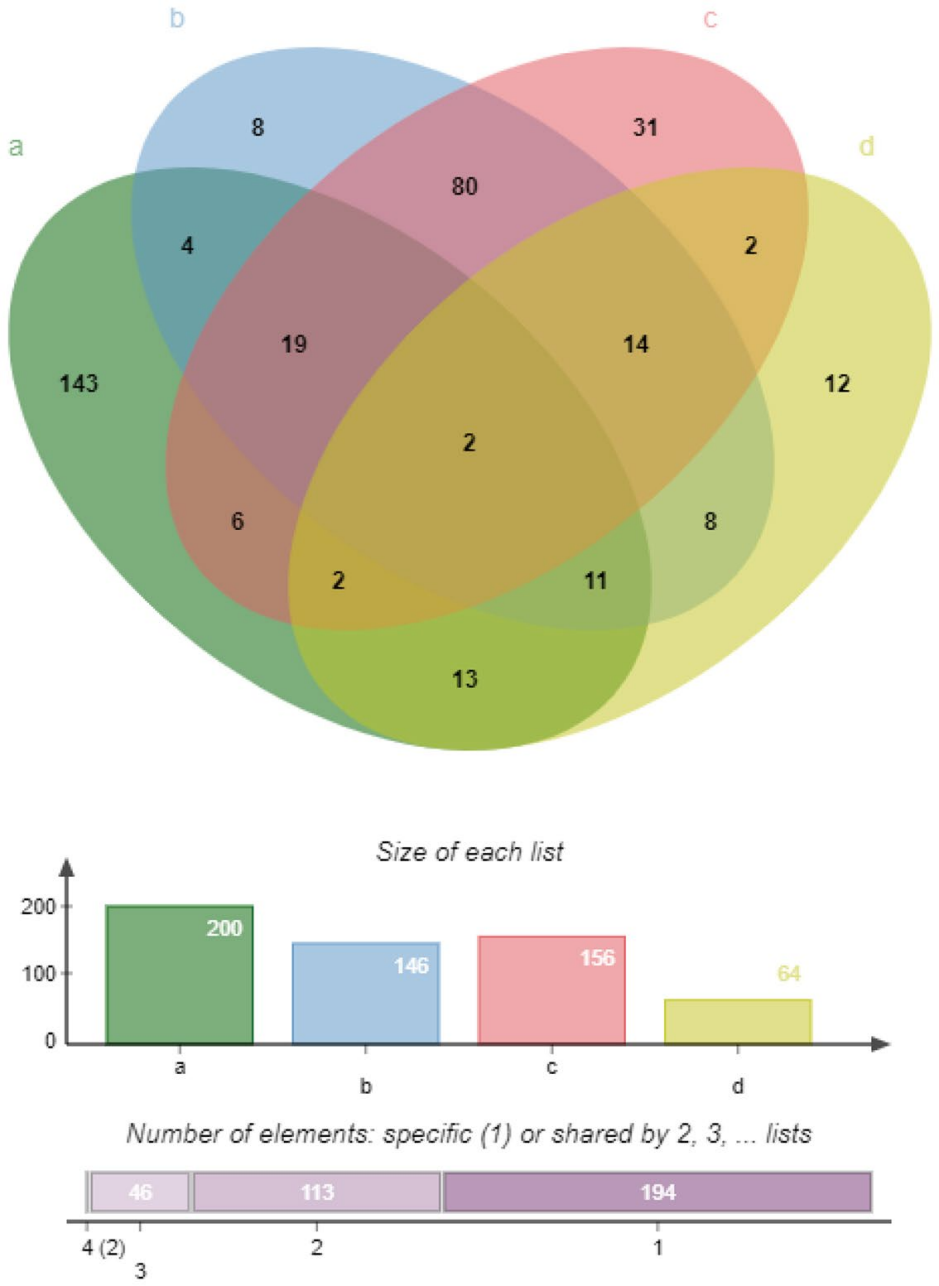
Venn diagram representing different classes of predicted membrane proteins, lipoproteins, and LAMPs. **(A)** LAMPs identified via LC MS/MS, green ellipse. **(B)** Membrane proteins identified via a proteomics approach based on Gram-negative predictive parameters, blue ellipse. **(C)** Membrane proteins identified based on Gram-positive predictive parameters, red ellipse. **(D)** Lipoproteins identified via LipoP – 1.0, yellow ellipse.

An analysis of the *M. synoviae* WVU1853 strain genome in the NCBI database revealed 673 protein-coding sequences (CDS), of which 10 were repeats. The remaining 663 proteins were subject to predictive analysis with a range of bioinformatics tools, identifying 136/663 (20.51%) as putative membrane proteins based on gram-negative predictive parameters (Supplementary Table 2). With respect to general localization, 112/663 (16.89%) samples were associated with the cytoplasmic membrane, 34/663 (5.13%) were associated with the outer membrane, and 7/663 (1.06%) were predicted to be extracellular (Table 1). The results of subcellular localization based on gram-positive predictive parameters (Supplementary Table 3). Of these proteins, 64 were identified as lipoproteins and 35 were located in the cell membrane (Supplementary Table 4). Membrane proteins identified via proteomic approach, putative lipoproteins via proteomic approach and LAMPs Venn diagram. According to the Gram-negative predictive parameters, 146 of our proteins were predicted as membrane proteins, 19 were considered to be predicted as lipoproteins, and 36 membrane proteins were detected by 92 mass spectrometry. According to Gram-positive predictive parameters, 156 proteins were predicted as membrane proteins, 20 membrane proteins were considered to be predicted as lipoproteins, and 29 membrane proteins were detected by mass spectrometry. Among 64 lipoproteins, 15 lipoproteins were detected in mass spectrometry (Figure 1).

**Table 1:** Subcellular distributions and numbers of proteins identified using Gram-negative bacterial predictive parameters.

	Location			
	Cytoplasmic membrane	Outer membrane	Extracellular	Others
Number	112	34	7	510

### Proteomic-based LAMP analysis

#### Analysis of *Mycoplasma synoviae* LAMPs

Proteomic analysis revealed that 41/200 (20.50%) of identified *M. synoviae* surface proteins were ribosomal proteins, 68/200 (34.00%) were enzymes, 11/200 (6.00%) were protein subunits, 10/200 (5.50%) were lipoproteins, 5/200 (2.50%) were elongation factors, 13/200 (6.50%) were hypothetical proteins, 51/200 (25.00%) other proteins (Supplementary Table 5). Of these proteins, 49 were identified as virulence-determining factors based on a BLAST score ≥ 80, while 2 proteins were considered cytotoxic proteins, 7 proteins were identified as neurotoxins, and 1 was identified as a predicted bacterial toxin (Supplementary Table 6). Twenty-one LAMPs were likely to contribute to increased pathogenicity following infection. One gene was considered a pathogenic effector gene while another was associated with susceptibility (Supplementary Table 7).

#### Data assessment and verification

**Figure 2:**
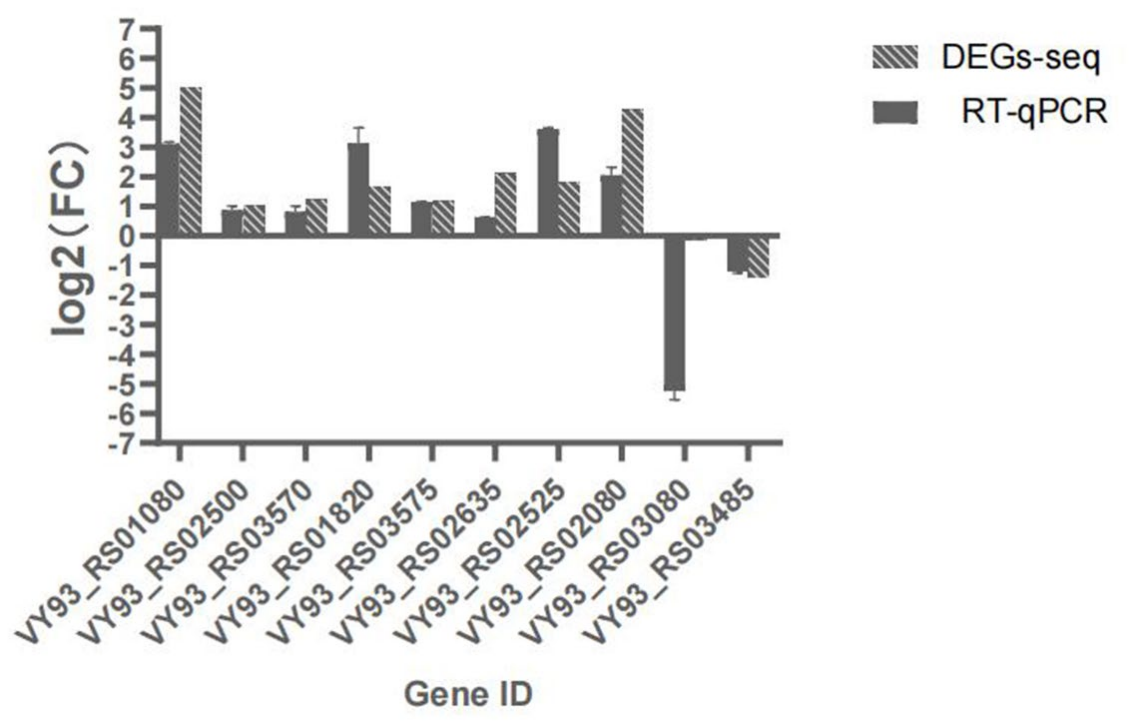
Expression of 10 DEGs as determined by RT-qPCR, the abscissa indicates Gene name，ordinate indicates log2(FC), Non-bar columns indicates RT-qPCR value, bar columns indicates corresponding genes log2(FC) value via transcriptome sequencing.

Ten LAMP genes were selected from the RNA-seq data and their expression verified by RT-qPCR. The RT-qPCR results showed same trends with the determination by RNA-seq, indicating the accuracy and quality of the RNA-seq results (Figure 2).

#### Differentially expressed LAMP gene identification

**Figure 3:**
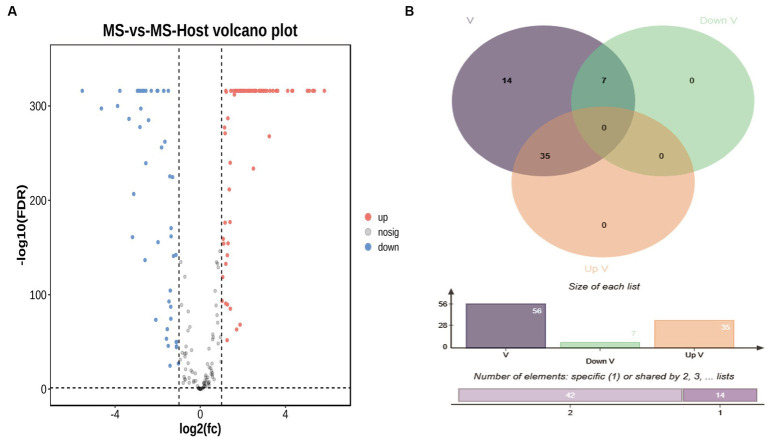
**(A)** Volcano plot representing differentially expressed genes (DEGs), Cutoff for log2(fc) is 1; the numbers of upregulated and downregulated genes were 72 and 47, respectively. **(B)** Venn diagram representing differentially expressed virulence factor genes. The blue circle represents virulence factors genes, the green circle represents down-regulated virulence factors genes, the red circle represents up-regulated virulence factors genes.

Differentially expressed LAMP genes (DELGs) were next analyzed, revealing that upon the infection of chicken cells changes in the LAMP transcriptome were evident (Supplementary Table 8), including the upregulation of 72 genes and the downregulation of 47 genes (Figure 3A). Forty-six genes predicted to encode virulence factors, 35 genes were upregulated and 7 genes were downregulated when *M. synoviae* exposed to host cells (Figure 3B). Furthermore, 7 and 4 genes associated with increased virulence were found to be upregulated and downregulated, respectively.

#### Go and pathway enrichment analysis

**Figure 4:**
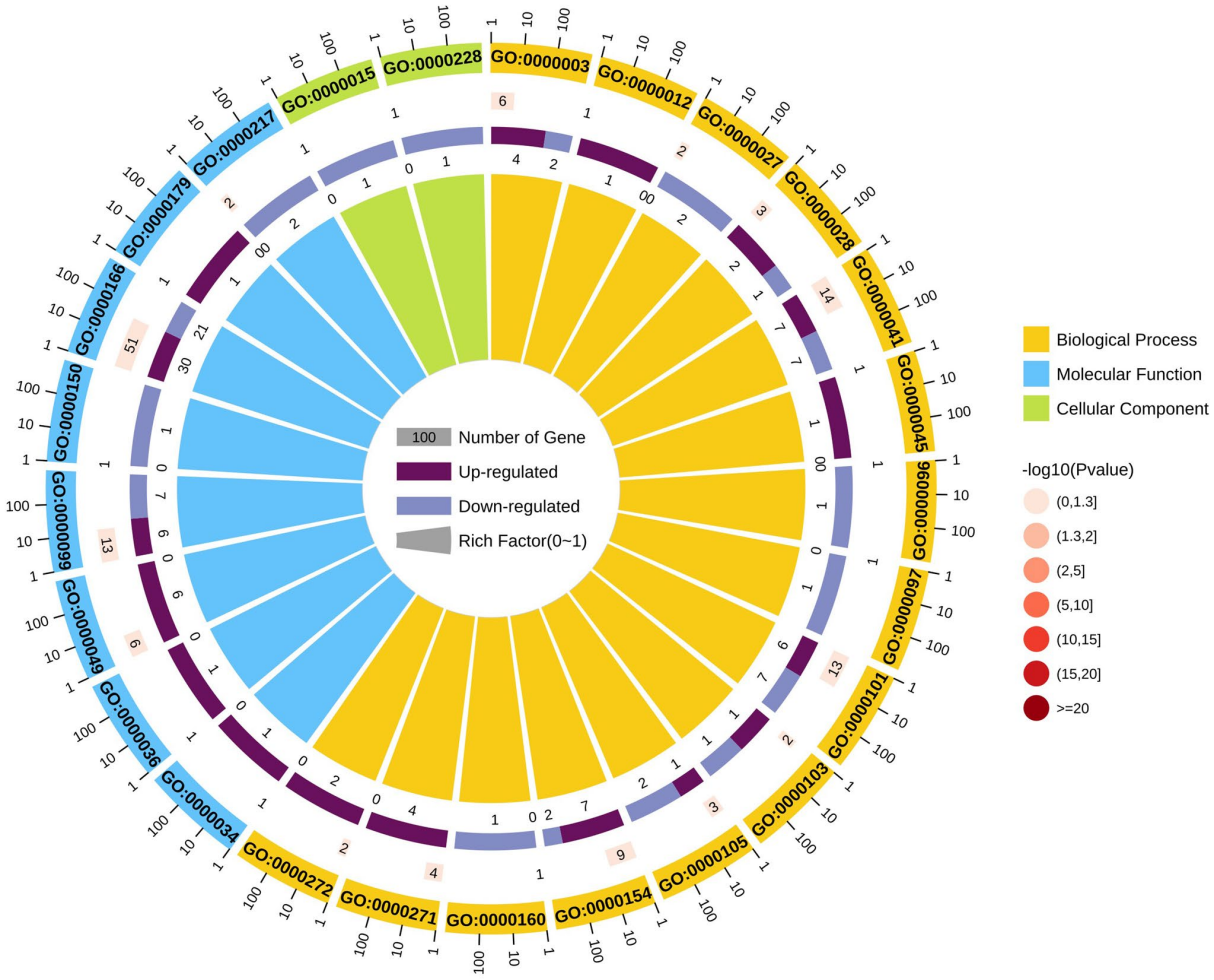
GO analysis of differentially expressed genes.

LAMP genes were categorized into the biological process (*n* = 64), cellular component (*n* = 2), and molecular function (*n* = 76) GO categories (Supplementary Table 9). Most differentially expressed genes associated with these GO categories were downregulated, and significant enrichment for the nucleotide binding pathway was observed. Moreover, GO terms enriched with upregulated DEGs (upregulated DEGs/total DEGs enriched in a GO category) among the top 25 GO categories included nucleotide binding (GO:0000166), sulfur amino acid transmembrane transporter activity (GO:0000099), and tRNA binding (GO:0000049). Most latent virulence factor and all pathogenic effector genes were enriched in the cellular component category (Figure 4).

**Figure 5:**
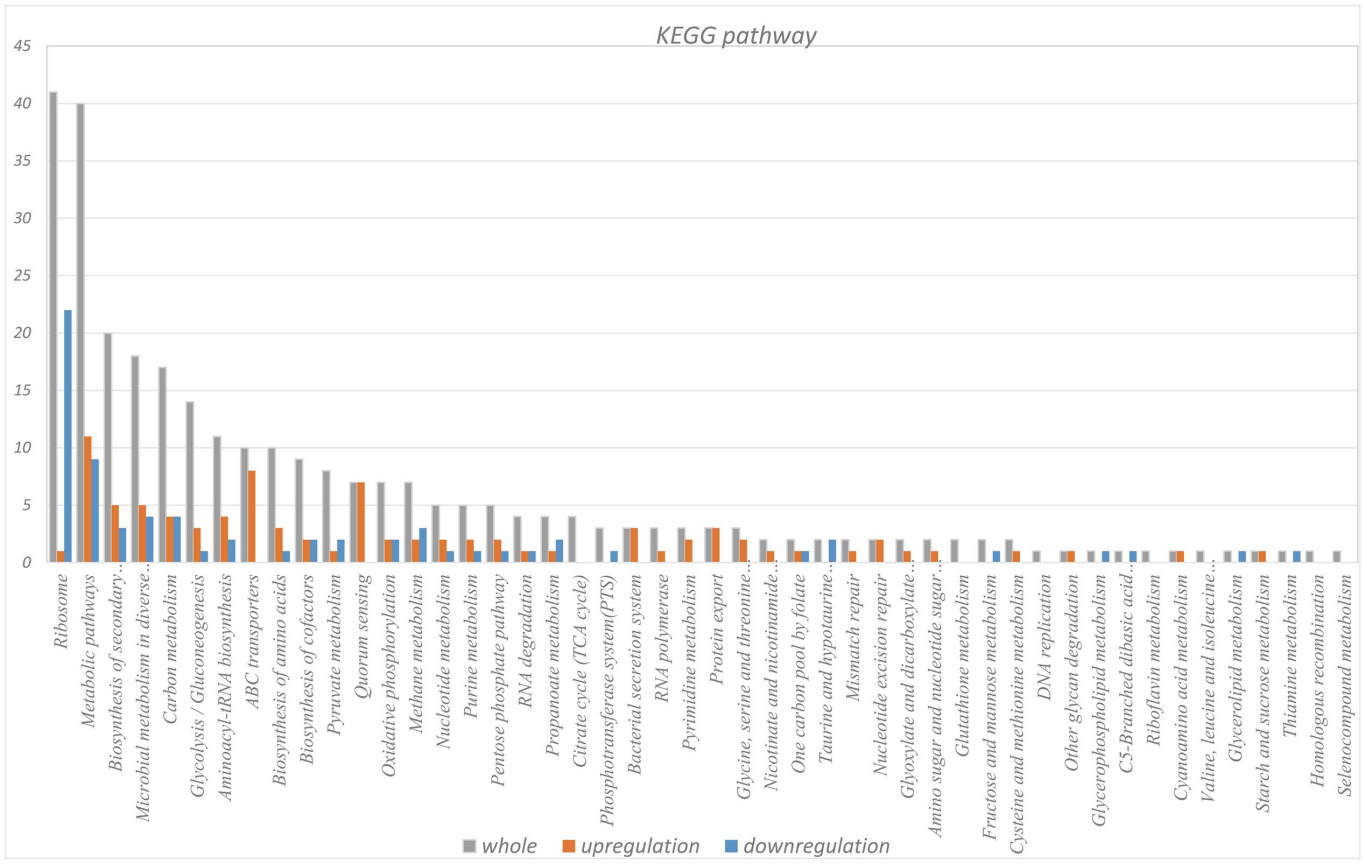
KEGG pathway analysis of differentially expressed gene.

**Figure 6:**
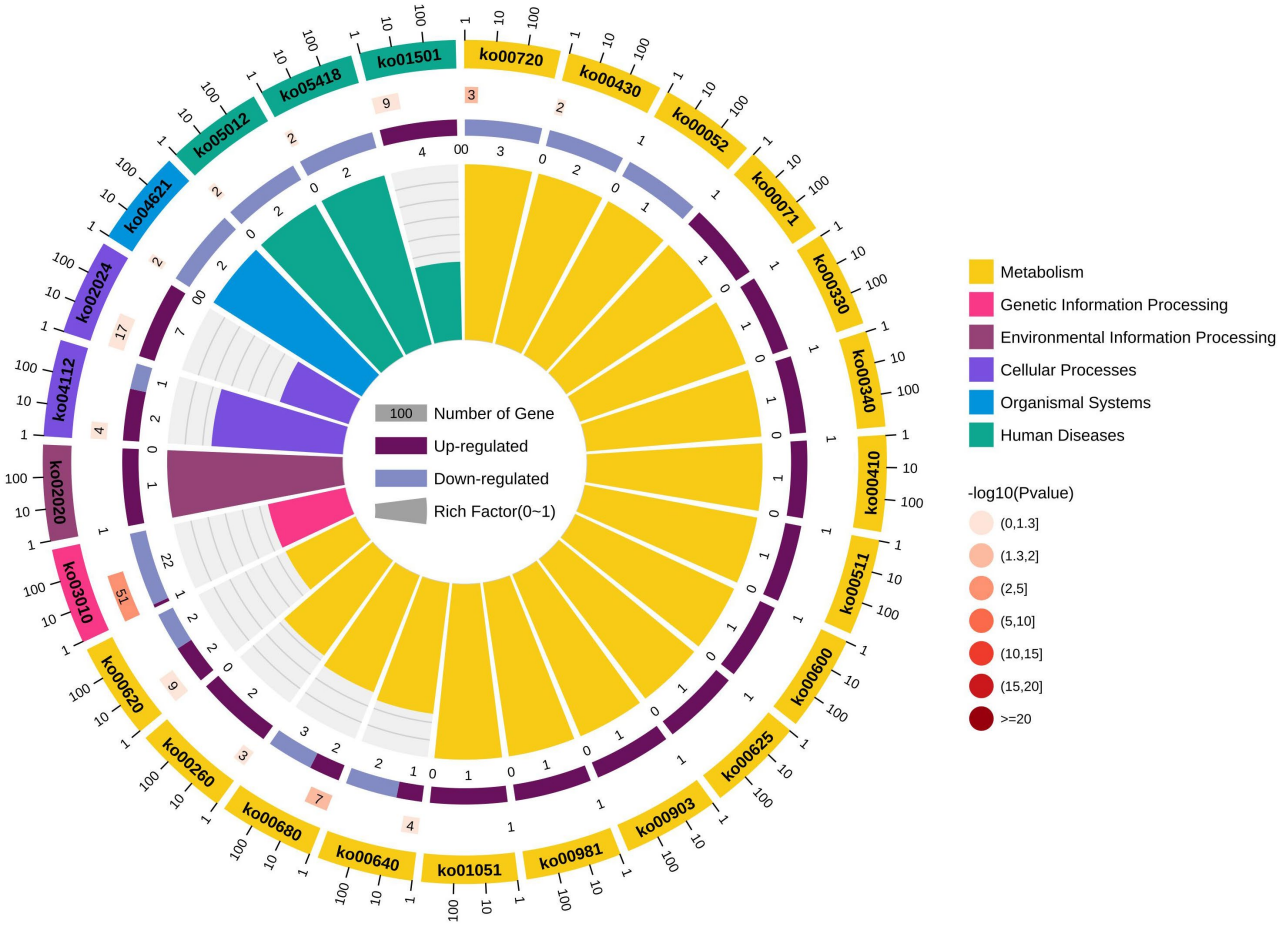
KEGG pathway differential enrichment circle chart.

KEGG pathway analysis of the identified LAMPs revealed their enrichment in 48 KEGG pathways (Supplementary Table 10). The ribosome pathway was most strongly enriched (41 genes), followed by the metabolic pathway (40 genes) and the biosynthesis of secondary metabolites pathway (20 genes). Other highly enriched pathways included the microbial metabolism in diverse environments, carbon metabolism, glycolysis/gluconeogenesis, aminoacyl-tRNA biosynthesis, biosynthesis of amino acids, ABC transporter, and biosynthesis of cofactors pathways which, respectively, contained 17, 16, 13, 11, 10, 10, and 8 genes, whereas all other enriched pathways were associated with fewer than 8 genes. In total, 25 putative virulence factor-encoding genes were subject to pathway enrichment analysis, with one gene (VY93_RS01495) being associated with 12 different pathways, while VY93_RS02635 was associated with 8 pathways, and VY93_RS03550 was associated with 7 pathways (Figure 5). Combing with histogram and circle chart, most of the differentially expressed genes associated with the ribosome pathway were downregulated, while the majority of genes associated with the quorum sensing and ABC transporter pathways were upregulated, differences in expression of other pathways were also clearly counted (Figure 6).

**Figure 7:**
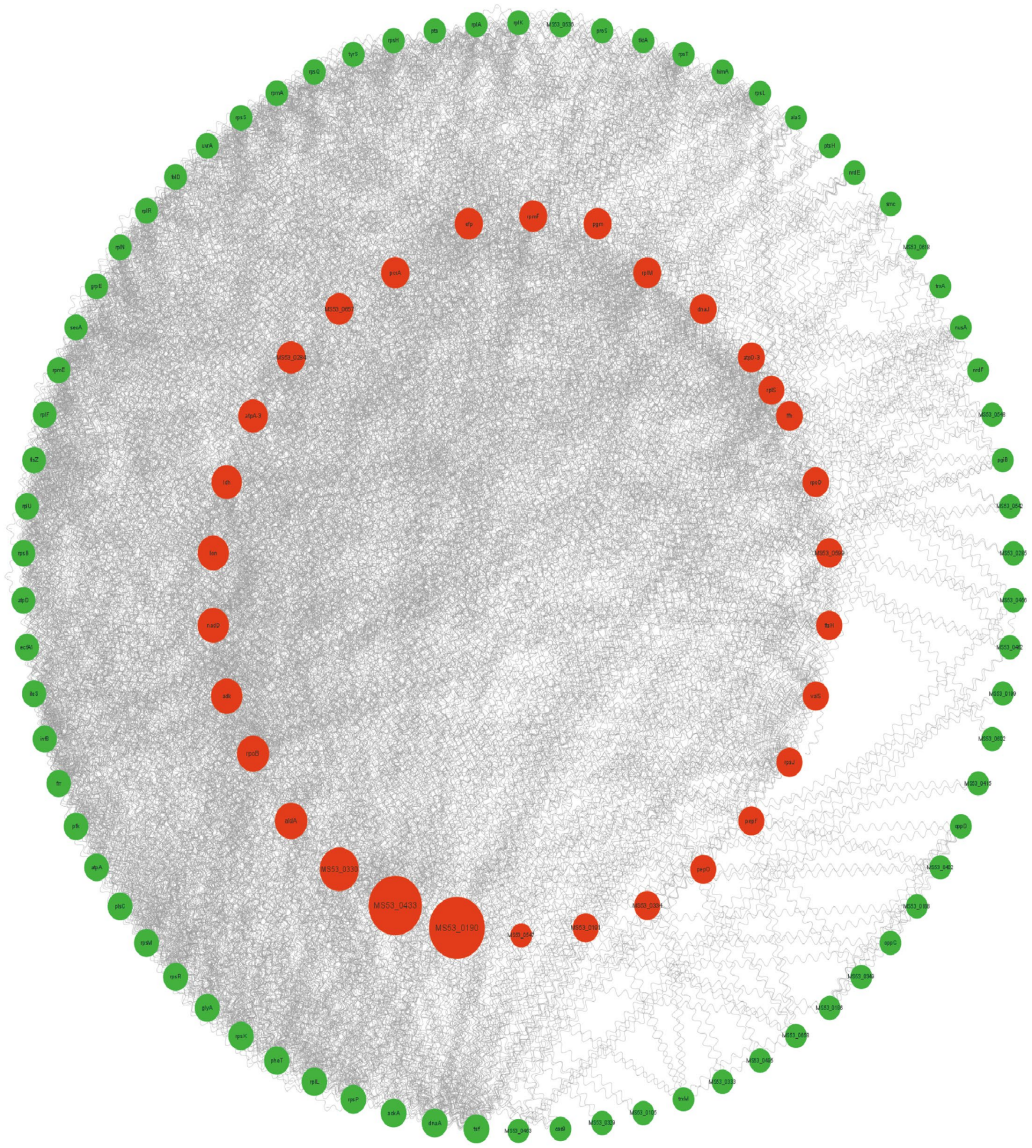
A protein–protein interaction network comprised of differentially expressed LAMP genes. Disconnected nodes are not displayed. Node size is proportional to betweenness centrality. Nodes in the inner circle are in the top 25%.

In total, 119 differentially expressed LAMPs were introduced into STRING to construct a PPI network comprised of 105 nodes and 895 edges, with the main clusters in this network covering 82.86% of nodes (Figure 7). Oligopeptide ABC transporters exhibited the highest level of betweenness centrality, interacting with 15 nodes.

## Discussion

*M. synoviae* is a devastating pathogen that can cause arthritis and synovitis in bird and poultry populations throughout the world. Owing to limited access to diagnostic tools, poor treatment options, and ineffective vaccines, the prevalence of *M. synoviae*-related disease continues to rise. LAMPs are a family of important virulence-associated proteins and potential immunogens that may offer utility as both diagnostic biomarkers and candidate targets for vaccine development. As *M. synoviae* do not possess a cell wall, their plasma membrane can closely interact with the membrane of host cells such that LAMPs are key targets for studies of host cell adhesion and invasion.

Here, an LC–MS/MS approach was used to comprehensively profile *M. synoviae* LAMPs, with predictive analysis of these LAMPs having additionally been performed prior to these analysis for subsequent comparison. Triton X-114 was used to extract lipoproteins, which were then detected and identified via LC–MS/MS as this is a standard approach used to explore the cell membrane-associated proteins for a range of pathogenic microbes (22, 23). *M. synoviae* LAMPs have not been the specific focus of prior research, and only 27 extracellular proteins have been defined for this pathogen (24). Several studies have employed 2D electrophoresis (2-DE) as a mean of identifying mycoplasma proteins following mass spectrometry (25, 26). However, these 2-DE strategies often overlook many membrane proteins. Signal peptide predictions can often be missed as a consequence of the misidentification of the methionine start site within the genome, as confirmed by the differences in the proportions of proteins localized into different compartments when using different analytical techniques. *M. synoviae* originates from gram-positive bacteria, but there are many differences between it and gram-positive bacteria, and gram stain of *M. synoviae* is negative (24, 27). Therefore, the subcellular localization of the proteins was predicted using two different parameters. The data obtained using the “gram-negative strain” parameter are closer to the experimental results. A total of 146 proteins were predicted as membrane proteins, 64 lipoproteins based on bioinformatics tool, fewer than LAMPs number detected by LC–MS/MS. When extracted LAMPs, fewer cytoplasmic proteins were mixed in, this results in a higher number of LAMPs detected. Certain LAMPs have been annotated in other organisms, supporting their potential identities as LAMPs in *M. synoviae*. By combining these data with proteomic results, the top 200 *M. synoviae* LAMPs were subject to further analysis.

Here, all the proteins, such as EF-Tu, that were mentioned in the introduction (apart from GDPD) were found. Through combinations of 12 functional analysis strategies including VFDB prediction, pathway, and conserved domain analysis, 49/200 proteins were identified as candidate virulence factors, of which 15 exhibited high BLAST scores for VFDB. Elongation factor Tu is a conserved cytoplasmic protein also unexpectedly localized on the surface of bacteria (28). Elongation factor Tu is the most abundant in bacterial total proteins, in this experiment it was identified as top 1 according to abundance, which confirmed that these proteomic analysis of *M. synoviae* LAMPs were reliable (29, 30). In our study, EF-Tu exhibited the highest BLAST score, with several conserved domains, and it may play a role in bacterial pathogenesis. EF-Tu is a multifunctional bacterial protein (31), and in *M. hyopneumoniae* it has been reported to protect against surface C3 deposition, binding to factor H and thereby preventing complement activation (32). Elongation factor Tu has been identified as an immunogenic membrane-associated protein that may contribute to *M. synoviae* pathogenesis. Cation-transporting P-type ATPases exhibited the second highest BLAST scores for VFDB with three independent domains, comprising a major family of membrane proteins present in eukaryotes, eubacteria, and archaea (33). The functions of cation-transporting P-type ATPases, however, have not been specifically explored in mycoplasma. Glucose-6-phosphate isomerase (GPI) is ubiquitously expressed across species and catalyzes reversible glucose-6-phosphate and fructose-6-phosphate isomerization in the glycolytic pathway (34). While *M. synoviae* GPI was not abundant on the cell surface, it was enriched in 8 pathways and predicted as a virulence factor with potential neurotoxin activity. The ABC transporter ATP-binding protein is an ABC transporter family member, and several proteins in this family have been established as virulence factors in addition to being ubiquitously expressed in bacteria, fungi, plants, mammals, and other species (35). Most of the proteins associated with increased virulence are enzymes. Of these, the ATP synthase subunit has been shown to be important for mycobacterial physiology and metabolism in *Mycobacterium smegmatis* (36), while in the *Panstrongylus megistus* ovary, β-ATPase functions as a docking lipophorin receptor. The role of β-ATPase also appears to be independent of its enzymatic ATP synthase activity (37).

When *M. synoviae* was exposed to host cell mixed cultures for 8 h, no significant upregulation of EF-TU, DnaK, or predicted virulence factor transcription was evident. All ATP synthase genes that are known to be involved in increased pathogenicity were observed to be upregulated which could suggest that the pathogenicity of the strain increases in response to host signals. VY93_RS01090, which produces the *Mycoplasma* and *Ureaplasma*-specific protein PDxFFG showed the most significant upregulation. However, the functional role of this protein remains to be determined. Exo-alpha-sialidase, which desialylates chicken IgGs and tracheal mucus glycoproteins, was also found to be upregulated in response to HD-11 and DF-1 cells (38). ABC transporter ATP-binding proteins are associated with multidrug resistance, and the *M. gallisepticum* Glycerol ABC Transporter has been reported to play a role in pathogenicity (39). The upregulation of two ABC transporter ATP-binding proteins (VY93_RS01085, VY93_RS01080) indicates that they may play a role in *M. synoviae* infection. Type III secretion system (T3SS) named protein-delivery machines was great targets for novel antimicrobial strategies (40). T3SS and their specific effectors are virulent and play an important in bacterial pathogenesis (41). Virulence reducing could help avoid recognition and attack by the host immune system, allowing pathogens survive in the host (42). Lipoprotein VY93_RS01160 is annotated as an *E. coli* type III secretion system translocator protein homolog, was significantly downregulated in this study. Active repression of the T3SS might contribute to the persistence of chronic infections, which may suggests the protein may be related to persistence of infections caused by *M. synoviae* (43). Quorum sensing plays an important role in virulence of other bacteria (44). When back-stimulated by host cells, bacteria communicate with each other to keep whole populations of bacteria alive (45, 46). In this experiment, seven genes (VY93_RS00130, VY93_RS00685, VY93_RS01075, VY93_RS01080, VY93_RS01085, VY93_RS03285, VY93_RS04195) enriched into quorum sensing pathway were upregulated, which may contribute to response certain factors released by host cells to enable *M. synoviae* survival.

In summary, the present study was developed to explore *M. synoviae* LAMP profiles using an LC–MS/MS approach. These results were used to guide the putative identification of the top 15 key LAMP-associated genes, and Elongation factor Tu was confirmed as a highly immunogenic LAMP. We offer new insight into the transcriptomic landscape of *M. synoviae* LAMPs in the context of *in vitro* host cell infection. Several proteins in the ABC transporter family were upregulated when these mycoplasma cells were exposed to chicken cells. How *M. synoviae* LAMPs play roles in cell adhesion, host immunity, apoptosis and other cell life processes requires further investigation. Together, these data may contribute to a more in-depth understanding of the pathogenesis of *M. synoviae* infections, offering an opportunity to establish candidate antigens for the development of appropriate vaccines aimed at preventing associated disease.

## Data availability statement

The original contributions presented in the study are included in the article/Supplementary materials, further inquiries can be directed to the corresponding authors.

## Ethics statement

Ethical approval was not required for the studies on animals in accordance with the local legislation and institutional requirements because only commercially available established cell lines were used.

## Author contributions

SH and JL designed the research and analyzed data. LG cultured the chicken cells. JL and FY participated in LAMPs extraction. DS prepared the figures and drafted the manuscript. DS, SH, and JL revised and approved the final manuscript. All authors contributed to the article and approved the version submitted for publication.

## Footnotes

^1^ https://www.psort.org/psortb/

^2^ http://www.cbs.dtu.dk/services/SecretomeP/

^3^ http://www.cbs.dtu.dk/services/SignalP/

^4^ http://www.cbs.dtu.dk/services/TMHMM/

^5^ https://services.healthtech.dtu.dk/service.php?LipoP-1.0

^6^ http://www.mgc.ac.cn/VFs/

^7^ https://www.omicshare.com/tools/Home/Soft/diffanalysis

^8^ https://www.omicshare.com/tools/Home/Soft/gogseasenior

^9^ http://www.kegg.jp/show_organism?menu_type=pathway_map&org=mbi

^10^ https://cn.string-db.org/
